# Patient‐Reported Oral Symptoms and Their Impact on Well‐Being After Haematopoietic Cell Transplantation

**DOI:** 10.1111/odi.70099

**Published:** 2025-09-18

**Authors:** Kristina Skallsjö, Michael T. Brennan, Bengt Hasséus, Jenny Öhman, Judith E. Raber‐Durlacher, Marie‐Charlotte D. N. J. M. Huysmans, Alexa M. G. A. Laheij, Stephanie J. M. van Leeuwen, Allan J. Hovan, Karin Garming Legert, Scott Isom, David M. Kline, Nicole M. A. Blijlevens, Jan‐Erik Johansson, Inger von Bültzingslöwen

**Affiliations:** ^1^ Department of Oral Medicine and Pathology, Institute of Odontology, The Sahlgrenska Academy University of Gothenburg Gothenburg Sweden; ^2^ Department of Oral Medicine/Oral & Maxillofacial Surgery Atrium Health Carolinas Medical Center Charlotte North Carolina USA; ^3^ Department of Otolaryngology/Head and Neck Surgery Wake Forest University School of Medicine Winston‐Salem North Carolina USA; ^4^ Department of Clinical Pathology Sahlgrenska University Hospital Gothenburg Sweden; ^5^ Department of Oral Medicine, Academic Centre for Dentistry Amsterdam University of Amsterdam and VU University Amsterdam the Netherlands; ^6^ Department of Oral and Maxillofacial Surgery, Amsterdam UMC University of Amsterdam Amsterdam the Netherlands; ^7^ Department of Dentistry Radboud University Medical Center Nijmegen the Netherlands; ^8^ Oral Oncology and Dentistry British Columbia Cancer Vancouver British Columbia Canada; ^9^ Department of Dental Medicine Karolinska Institutet Huddinge Sweden; ^10^ Department of Biostatistics and Data Science, Division of Public Health Sciences Wake Forest University School of Medicine Winston‐Salem North Carolina USA; ^11^ Department of Hematology Radboud University Medical Center Nijmegen the Netherlands; ^12^ Department of Hematology and Coagulation, The Sahlgrenska Academy University of Gothenburg Gothenburg Sweden; ^13^ Department of Oral Microbiology and Immunology, Institute of Odontology, The Sahlgrenska Academy University of Gothenburg Gothenburg Sweden

**Keywords:** adverse effects, haematopoietic cell transplantation, mouth symptoms, oral complications, patient‐reported outcomes, quality of life

## Abstract

**Objective:**

Oral complications may negatively influence outcomes of haematopoietic cell transplantation (HCT). A comprehensive view of oral symptoms and symptom burden post‐HCT is lacking. This study aimed to determine the prevalence, severity, and temporal relationships of oral symptoms and their impact on well‐being in the early post‐HCT phase. Effects of transplant type and conditioning intensity were evaluated.

**Methods:**

In this prospective multicentre observational study, adult HCT recipients were interviewed and completed questionnaires on oral symptoms and well‐being three times a week during hospitalisation early post‐HCT.

**Results:**

Of 194 patients, 177 (91.2%) reported oral symptoms. Dry mouth was the earliest and most common (80.9%) followed by oral pain (35.6%), thickening/swollen mucosa (33.0%), and taste changes (30.9%). Symptom frequency peaked on days 6 to 11 post‐HCT and caused significant burden: 59.3% experienced moderate to severe distress and 53.6% reported moderate to severe impact on well‐being. Symptom prevalence was highest among patients who received allogeneic HCT with MAC and those who underwent autologous HCT. Overall, MAC regimens were associated with earlier and more frequent symptoms, greater distress and higher impact on well‐being during days 0 to 11 post‐HCT compared to reduced/non‐myeloablative regimens.

**Conclusions:**

Oral symptoms are prevalent, burdensome and significantly impact well‐being early post‐HCT, underscoring the need for close monitoring and supportive oral care.

## 1 Introduction

Haematopoietic cell transplantation (HCT) is a potentially curative treatment for patients with haematological malignancies as well as for certain non‐malignant diseases such as primary immunodeficiencies and aplastic anaemia (Copelan 2006). In autologous HCT, myeloablative conditioning (MAC) is the standard regimen whereas in allogeneic HCT, MAC, reduced‐intensity conditioning (RIC) or non‐myeloablative conditioning (NMA) is selected depending on the clinical context (Bacigalupo et al. 2009; Gyurkocza and Sandmaier 2014).

During the first weeks after HCT, patients often experience a multitude of treatment‐related side effects and symptoms which may significantly affect both quality of life and treatment outcomes (El‐Jawahri et al. 2015; Garcia et al. 2012; Larsen et al. 2004). There is a limited understanding of the scope and burden of oral symptoms in the early post‐HCT period. Oral mucositis, mucosal infections, pain, xerostomia (dry mouth), taste changes, and swallowing difficulties are some of the commonly encountered symptoms that can add to the overall symptom burden (Agholme et al. 2023; Bos‐den Braber et al. 2015; Elad et al. 2015). Xerostomia and oral pain related to oral mucositis have been described as some of the most distressing symptoms reported by HCT recipients (Bellm et al. 2000; Larsen et al. 2004). These and other symptoms not only compromise oral comfort and health, but can also interfere with basic daily activities, the ability to maintain adequate nutrition, and delay or compromise recovery after HCT.

Although various oral complications have been reported during the first weeks after HCT (Bos‐den Braber et al. 2015; Elad et al. 2015; Epstein et al. 2009; Wysocka‐Słowik et al. 2021), less is known about the collective overall picture of all oral symptoms including temporal relations and the burden of these symptoms on patients' overall well‐being. Also, studies suggest that RIC and NMA conditioning regimens are associated with fewer and less severe symptoms and a lower symptom burden compared to MAC, but the evidence is not consistent (Andersson et al. 2009; El‐Jawahri et al. 2015; Wysocka‐Słowik et al. 2021).

In order to identify oral symptoms that can be important targets for intervention and improved management, a better understanding of patients' experiences of oral symptoms after HCT is essential. While some symptoms are difficult to avoid, it may be possible to treat and relieve symptoms with supportive care measures. This may in turn prevent secondary effects or the development of other symptoms that share a common aetiology. The aim of this prospective observational study was to determine the prevalence, severity, and temporal relationship of patient‐reported oral symptoms in the early phase following HCT as well as their impact on well‐being. Additionally, the effects of transplant type and conditioning intensity were evaluated.

## 2 Materials and Methods

Patients ≥ 18 years old scheduled for autologous or allogeneic HCT were included in this observational multicentre study (the Orastem study). Patients were enrolled at the following sites starting in March 2011 and continuing at intervals until May 2018: Sweden: Sahlgrenska University Hospital, Gothenburg and Karolinska University Hospital Huddinge, Stockholm, Sweden; Atrium Health Carolinas Medical Center, Charlotte, NC, USA; BC Cancer, Vancouver, BC, Canada; Amsterdam UMC, University of Amsterdam and Radboud University Medical Center, Nijmegen, The Netherlands. Written informed consent was obtained from all patients included in the study. A complete description of the Orastem study protocol has been previously published (Brennan et al. 2018).

Bedside visits by dental personnel during hospitalisation were scheduled three days/week after transplantation until the absolute neutrophil count (ANC) reached > 0.5 × 10^9^/L. Beyond this time, patients with continued oral mucositis or other oral problems requiring hospitalisation were scheduled for oral examinations three days/week for up to six weeks. At each visit, patients were interviewed about their oral symptoms according to a set of standardised open‐ended questions predefined in the Orastem study protocol (Brennan et al. 2018). To reduce inter‐examiner variability, all examiners were trained and calibrated in the measurements used in the study. Patients were asked if they experienced any oral symptoms, the nature of their symptoms, and their worst oral symptoms. Patients were also asked to grade how much any particular symptom bothered them using a 0–10‐point numerical rating scale (NRS).

In addition to the interviews, patients were asked to fill in a questionnaire three times/week about their oral symptoms. Two questions concerned how the oral symptoms affected their general well‐being: (i) how much distress the oral symptoms caused (“During the past 24 h, how much did the overall feeling of your mouth distress or bother you?”) and (ii) how much the oral cavity symptoms affected the patient's well‐being (“During the past 24 h, how much did the overall feeling of your oral cavity affect your well‐being?”). Both questions were answered using a five‐point Likert‐type scale (0 = not at all, 1 = a little, 2 = some, 3 = a lot, 4 = severely).

Medical diagnosis, conditioning regimen, transplantation type, and white blood cell (WBC) count (including ANC) were retrieved from the medical records. The intensity of the conditioning regimen was classified as MAC, RIC, or NMA by the haematologists at each centre based on the expected duration of cytopenia and requirement for stem cell support (Bacigalupo et al. 2009). The classifications were reviewed by two haematologists (JEJ and NB).

### 2.1 Statistical Analyses

For the analyses, patients were included if they had responded to at least one oral symptom question during the interviews and had completed one questionnaire. Frequency counts and proportions were calculated to describe the prevalence of oral symptoms during hospitalisation. For analyses of temporal relationships, patient‐reported oral symptoms were categorised by days after HCT since bedside visits were not performed each day. The following time intervals were chosen, starting day 0 (the day of HCT): days 0 to 5; 6 to 11; 12 to 17. For patients with multiple observations during a time interval, the worst patient response (symptom ever present, distress/impact on wellbeing) was used so that each patient is only represented once during a period. Past day 17 after HCT, only one‐third of the patients continued to be followed within this part of the study (two‐thirds of the patients had ANC > 0.5 × 10^9^/L). Therefore, to achieve statistically robust conclusions, the results are focused on days 0 to 17 post‐HCT. Time to first event plots showed when each oral symptom was first reported post‐transplant and presented as cumulative incidence during hospitalisation. As patients were not assessed daily, the data were interval‐censored, with symptom onset assumed to occur between the last negative and first positive report. In the time to first event analyses, two patients were excluded because they were not seen during the first week.

Generalised log‐rank tests with constant weights were used to measure differences between the conditioning regimen intensity groups. The daily probability of symptoms was modelled with mixed effects logistic regression models with thin plate splines to estimate the effect of days after HCT across all patients and within each conditioning regimen intensity group. Each model was adjusted for site, age, and sex and included a random intercept for patient. The results from the two Swedish sites were combined in the analyses due to a limited number of included patients in one of the sites. Both Swedish centres followed the same treatment regimens. Association of symptom occurrence (both any symptom and each specific symptom) and measures of well‐being were investigated by chi‐square tests and then mixed effects logistic regression models, adjusting for clinical site, regimen intensity (MAC vs. RIC/NMA), transplant type, sex, age, and days since HCT.

## 3 Results

A total of 195 patients in the Orastem study (Skallsjö et al. 2023) were interviewed bedside and asked to fill in the questionnaires during hospitalisation after HCT. One patient was unable to respond due to severe illness, was admitted to intensive care and died shortly after transplantation. Thus, the study cohort consisted of 194 patients. The clinical characteristics of the patients are presented in Table 1. Of the 121 (62.4%) patients undergoing allogeneic HCT, 92 (76.0%) patients were treated with RIC or NMA, and 29 (24.0%) patients received MAC. Among the 73 autologous HCT recipients, all except three received MAC (95.9%).

**TABLE 1:** Clinical characteristics of the cohort by type of transplantation.

	Full cohort (*N* = 194)	Allogeneic (*N* = 121)	Autologous (*N* = 73)
Age, median (min; max)	56.0 (18.0; 76.0)	54.0 (18.0; 76.0)	57.0 (30.0; 69.0)
Sex			
Male	107 (55.2%)	67 (55.4%)	40 (54.8%)
Female	87 (44.8%)	54 (44.6%)	33 (45.2%)
Medical diagnosis			
Acute myeloid leukaemia	45 (23.2%)	45 (37.2%)	0
Acute lymphocytic leukaemia	13 (6.7%)	13 (10.7%)	0
Lymphoma	26 (13.4%)	18 (14.9%)	8 (11.0%)
Chronic lymphocytic leukaemia	5 (2.6%)	5 (4.1%)	0
Myelodysplastic syndrome	14 (7.2%)	14 (11.6%)	0
Chronic myeloid leukaemia	5 (2.6%)	5 (4.1%)	0
Myelofibrosis	10 (5.2%)	10 (8.3%)	0
Severe aplastic anaemia	4 (2.1%)	4 (3.3%)	0
Multiple myeloma	62 (32.0%)	1 (0.8%)	61 (83.6%)
Other^a^	10 (5.2%)	6 (5.0%)	4 (5.5%)
Treatment intensity			
Myeloablative conditioning (MAC)	99 (51.0%)	29 (24.0%)	70 (95.9%)
Reduced intensity conditioning (RIC)	77 (39.7%)	74 (61.2%)	3 (4.1%)
Non‐myeloablative conditioning (NMA)	18 (9.3%)	18 (14.9%)	0
Conditioning regimen			
BEAM	9 (4.6%)	0	9 (12.3%)
Busulfan/Fludarabine	24 (12.4%)	24 (19.8%)	0
Busulfan/Cyclophosphamide	3 (1.5%)	3 (2.5%)	0
Cyclophosphamide	6 (3.1%)	5 (4.1%)	1 (1.4%)
Cyclophosphamide/TBI	18 (9.3%)	18 (14.9%)	0
Fludarabine/TBI	18 (9.3%)	18 (14.9%)	0
Fludarabine/TBI/Cyclophosphamide	42 (21.6%)	42 (34.7%)	0
Fludarabine/Treosulfan	4 (2.1%)	4 (3.3%)	0
Melphalan 140	2 (1.0%)	0	2 (2.7%)
Melphalan 200	62 (32.0%)	1 (0.8%)	61 (83.6%)
Other^b^	6 (3.1%)	6 (5.0%)	0

Abbreviations: BEAM, Carmustine, Etoposide, Cytarabine, Melphalan; FLAMSA, Fludarabine, Amsacrine, Cytarabine; POEMS syndrome, Polyneuropathy, Organomegaly, Endocrinopathy, M‐protein, Skin changes‐syndrome; TBI, Total Body Irradiation; TLI‐ATG, Total Lymphoid Irradiation and AntiThymocyte Globulin.

^a^ Sickle cell anaemia *n* = 3, POEMS syndrome *n* = 2, Prolymphocytic leukaemia *n* = 1, Paroxysmal nocturnal haemoglobinuria *n* = 1, Haemophagocytic lymphohistiocytosis *n* = 1, Multiple sclerosis *n* = 1, Chronic inflammatory demyelinating polyneuropathy *n* = 1.

^b^ Other include Fludarabine/Melphalan *n* = 2; TBI + Monoclonal antibody *n* = 1; FLAMSA *n* = 2; TLI‐ATG *n* = 1.

### 3.1 Oral Symptoms During Hospitalisation Post‐HCT

On days 0 to 17 post‐HCT, 177 (91.2%) of the 194 patients reported at least one oral symptom and more than two‐thirds of patients (68.0%) experienced multiple symptoms (≥ 2) (Table 2). The most frequent oral complaint was dry mouth (80.9%) followed by oral pain (35.6%), thickening/swollen mucosa (33.0%) and taste changes (30.9%). Overall, days 6 to 11 was the interval with the highest proportions of patients reporting symptoms, coinciding with the nadir of WBC/ANC. On their worst day, patients reported an average of 2.3 (SD 1.6) simultaneous symptoms throughout the first 17 days of hospitalisation post‐HCT (Table 2). By day 12 to 17, “any oral symptom” persisted in two‐thirds of the total cohort. The decrease in proportion of symptoms was due to a statistically significant reduction of symptoms in patients undergoing autologous HCT (data not shown).

**TABLE 2:** Frequency and timing of symptoms by days after haematopoietic cell transplantation (HCT).

	Total (Days 0–17)	Day 0–5	Day 6–11	Day 12–17
	*N* = 194	*N* = 189	*N* = 186	*N* = 173
Any oral symptom (total)	177 (91.2)	135 (71.4)	159 (85.5)	113 (65.3)
Dry mouth	157 (80.9)	112 (59.3)	112 (60.2)	87 (50.3)
Oral pain	69 (35.6)	26 (13.8)	55 (29.6)	35 (20.2)
Thickening of mucosa/swollen mucosa	64 (33.0)	31 (16.4)	48 (25.8)	20 (11.6)
Taste change	60 (30.9)	36 (19.0)	35 (18.8)	30 (17.3)
Mucosal sensitivity	51 (26.3)	21 (11.1)	35 (18.8)	14 (8.1)
Sticky saliva	51 (26.3)	21 (11.1)	34 (18.3)	17 (9.8)
Sensitive/tender teeth	28 (14.4)	6 (3.2)	18 (9.7)	9 (5.2)
Mucosal coating	13 (6.7)	5 (2.6)	9 (4.8)	2 (1.2)
Easy bleeding from gums/oral mucosa	13 (6.7)	4 (2.1)	9 (4.8)	3 (1.7)
Bad breath (halitosis)	3 (1.5)	1 (0.5)	1 (0.5)	2 (1.2)
Other oral symptoms^a^	53 (27.3)	18 (9.5)	36 (19.4)	22 (12.7)
Multiple oral symptoms (≥ 2)	132 (68.0)	80 (42.3)	101 (54.3)	66 (38.2)
Mean no. symptoms (SD)	2.3 (1.6)	1.4 (1.3)	1.9 (1.6)	1.3 (1.4)

*Note:* Patients could have more than one event in each time interval. Data are number (%) of patients reporting symptoms unless stated otherwise.

^a^ Other oral symptom, not specified.

After day 17, approximately one‐third of patients (*n* = 60) continued to be followed in accordance with the study protocol. Of these, 47/60 (78.3%) had received allogeneic HCT. This proportion was significantly higher compared to those patients followed only up to day 17 (*p* = 0.0021).

### 3.2 Time to First Event of Oral Symptoms

**FIGURE 1:**
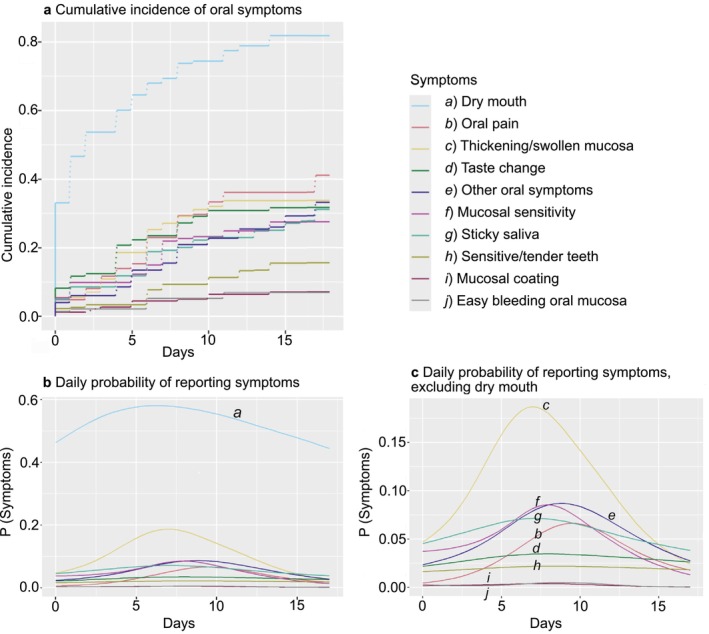
(a) Cumulative incidence of oral symptoms related to time after haematopoietic cell transplantation (HCT). (b) Mixed effects models showing the daily probability of reporting each of the symptoms by day of hospitalisation during the first 17 days post‐HCT. The top ten symptoms are shown in the model. (c) Same as (b) but excluding dry mouth. Models are adjusted for age, sex and site.

A majority of patients reported that oral symptoms appeared within the first week after transplantation. By day 4 after transplantation, 63.0% (*n* = 121) had experienced oral symptoms. By day 6, 79.7% (*n* = 153) had experienced oral symptoms. Time to first symptom plots comparing all individual symptoms showed that the cumulative incidence of dry mouth was 40% to 50% within the first few days after HCT while the cumulative incidence of the other oral symptoms was lower (Figure 1a).

### 3.3 Daily Probability and Temporal Relationship of Oral Symptoms

The daily probability of reporting oral symptoms during the first 17 days after HCT was explored in mixed effects models (Figure 1b,c). The peak probability for each specific symptom was reached at various time points after HCT, with dry mouth presenting the highest overall probability by day 6, followed by thickening/swollen oral mucosa. For oral pain, the highest probability was seen by day 9 to 10 post‐HCT (Figure 1b,c).

### 3.4 Worst Oral Symptom

Dry mouth, oral pain and taste changes were reported as the worst oral symptoms during hospitalisation (Table S1). Overall, patients rated their worst symptoms on an NRS with the highest mean scores recorded for oral pain (5.7, SD 2.4), taste change (5.3, SD 3.0), dry mouth (5.2, SD 2.4) and sticky saliva (5.2, SD 3.2). The highest NRS scores per patient were reported in the time interval 6 to 11 days after HCT.

### 3.5 Oral Symptoms Related to Transplant Type and Conditioning Intensity

Symptoms were most common in patients receiving MAC: 100% (*n* = 29) in allogeneic HCT with MAC and 94.3% (*n* = 66) in autologous HCT with MAC, compared to 85.9% (*n* = 79) in allogeneic HCT with RIC/NMA (Table S2). In the allogeneic MAC group, symptom prevalence remained consistently high across all intervals: days 0 to 5 (89.7%), days 6 to 11 (89.7%), and days 12 to 17 (84.0%). While both groups treated with MAC showed similarly high symptom frequency on days 6 to 11 (autologous: 92.6%, allogeneic: 89.7%), symptoms declined in the autologous group by days 12 to 17 (58.7%). Those undergoing allogeneic transplant with RIC/NMA reported lower symptom prevalence than the allogeneic MAC group at all time points (Table S2). The impact of conditioning intensity on oral symptoms was further analysed in time to first symptom plots and probability models, comparing MAC (allogeneic and autologous HCT) with RIC and NMA.

**FIGURE 2:**
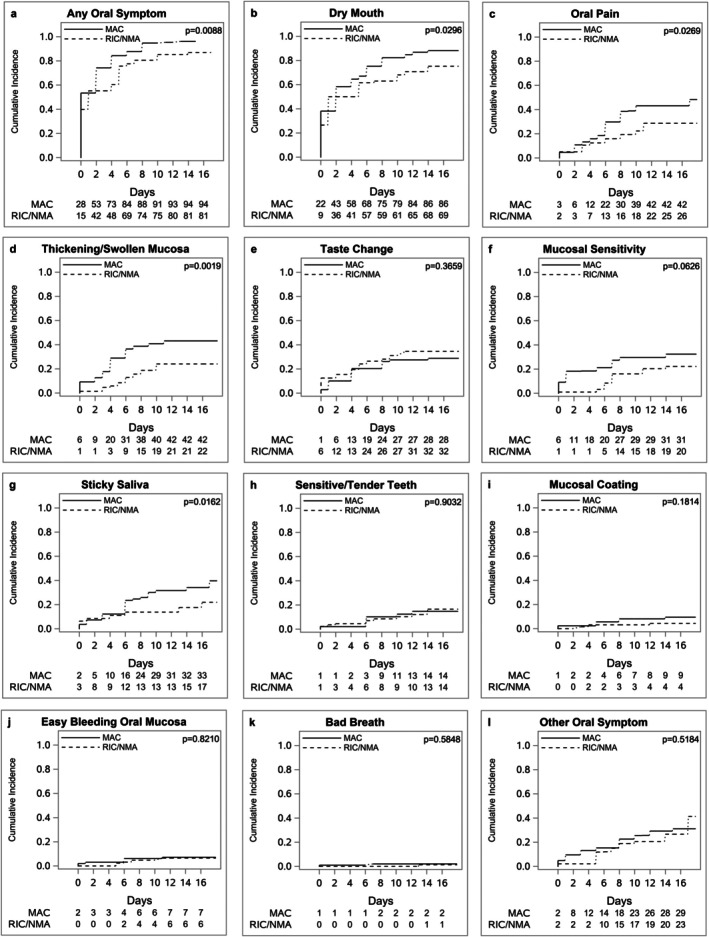
Cumulative incidence of patient‐reported oral symptoms during days 0 to 17 following haematopoietic cell transplantation (HCT), showing (a) any symptom and (b–l) individual symptoms over time. Time to first reported symptom is compared by conditioning regimen intensity: Myeloablative conditioning (MAC) versus reduced intensity (RIC), or non‐myeloablative conditioning (NMA). Numbers shown below the plots represent cumulative counts of events by day following HCT. Statistical differences were assessed using log‐rank tests.

Time to first symptom plots showed that “any oral symptom” (*p* = 0.0088), dry mouth (*p* = 0.0296), oral pain (*p* = 0.0269), thickening/swollen mucosa (*p* = 0.0019) and sticky saliva (*p* = 0.0162) appeared earlier and with a higher cumulative incidence in patients treated with MAC compared to RIC/NMA (Figure 2).

**FIGURE 3:**
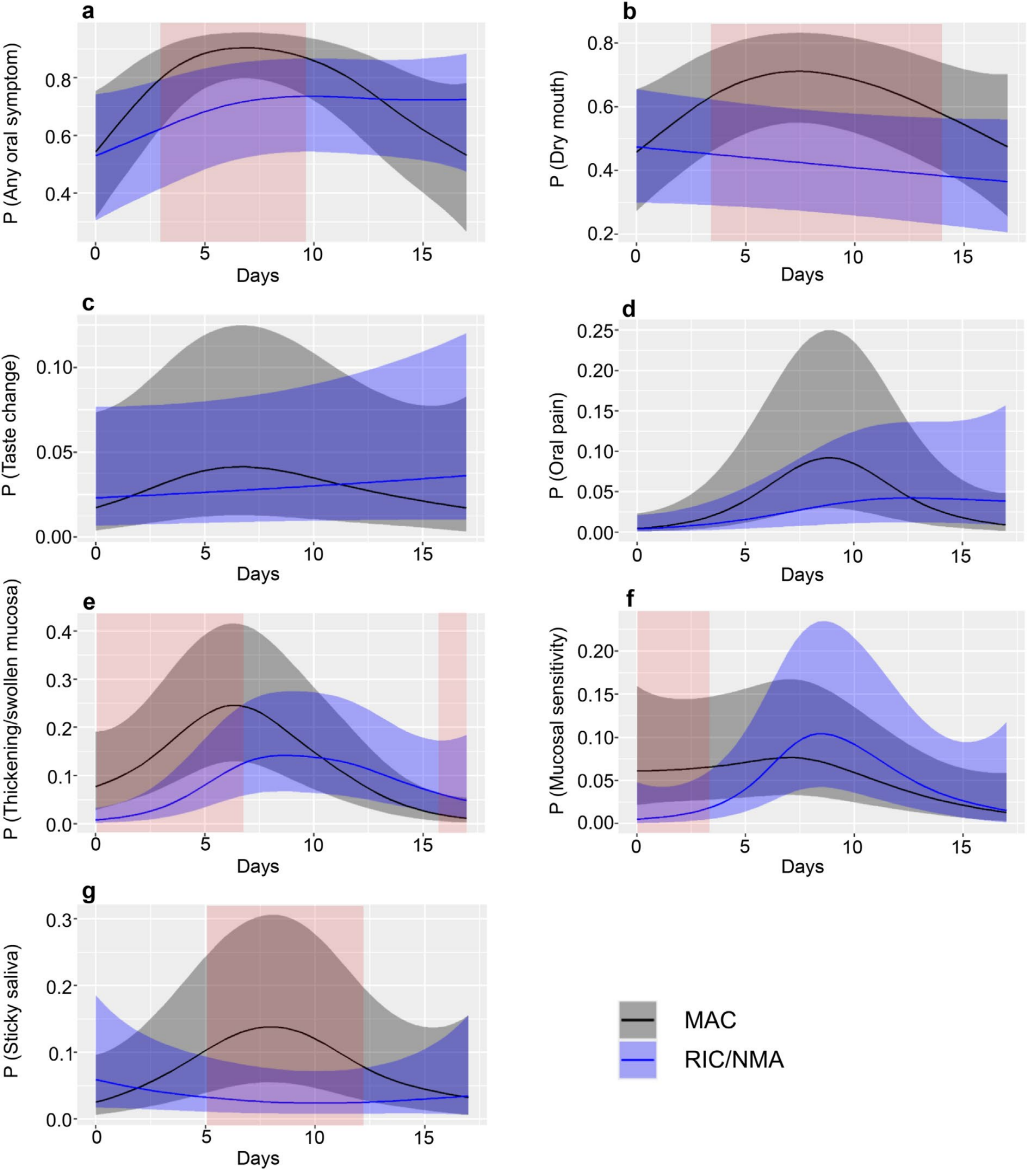
Daily probability of reporting (a) any oral symptom, and (b–g) specific oral symptoms day 0 to 17 after haematopoietic cell transplantation (HCT), comparing patients who received myeloablative conditioning (MAC; black line) with those who received reduced‐intensity or non‐myeloablative conditioning (RIC/NMA; blue line) with shaded areas representing 95% confidence intervals. Red shading indicates periods with significant differences between the two groups (*p* < 0.05). Symptoms with a prevalence of 15% or lower are not displayed. All models are adjusted for age, sex, and site.

The daily probability of experiencing oral symptoms was higher in patients treated with MAC for several of the specific symptoms investigated. Significant differences were observed for “any oral symptom”, dry mouth, thickening/swollen mucosa, mucosal sensitivity and sticky saliva during certain time periods after HCT, as shown by red shading in Figure 3. Data not shown for mucosal coating, sensitive teeth and other symptoms (differences were non‐significant between the intensity groups).

### 3.6 Symptom Burden

For analyses of patient‐reported symptom burden, questionnaire answers 0 to 1 (not at all or a little) were considered as none or low symptom burden while answers ranked 2 to 4 (some, a lot, or severe) were categorised as moderate to severe symptom burden. Among the 177 patients who reported any oral symptom during hospitalisation, 59.3% (*n* = 105) answered that the overall feeling of their mouth caused them moderate to severe distress. Furthermore, 53.6% (*n* = 95) of patients reported that the overall feeling of the mouth had a moderate to severe impact on their well‐being. The proportion of patients reporting moderate to severe symptom burden (distress and impact on well‐being) related to the overall feeling of the mouth peaked in the time interval 6 to 11 days after HCT.

Mixed effects logistic regression models showed that each of the patient‐reported oral symptoms was statistically significantly associated with increased distress and worse well‐being due to the overall feeling of the mouth (Table 3). The only exception were symptoms categorised as “other oral symptom” (i.e., symptom not specified) (Table 3). Among the individual symptoms, the strongest association was seen for oral pain (12.3 times greater odds of experiencing worse distress and 19.5 times greater odds for worse well‐being) compared to patients who did not have oral pain (Table 3). Multiple oral symptoms were associated with worse symptom burden shown by the fact that for every additional symptom, patients had increased odds (OR 2.7, *p* < 0.0001) of experiencing worse distress and 2.6 times greater odds (*p* < 0.0001) of having worse well‐being (Table 3).

**TABLE 3:** The impact of oral symptoms on symptom burden after haematopoietic cell transplantation (HCT).

Oral symptom	During the past 24 h, did the overall feeling of your mouth distress or bother you ‘some/a lot/severely’?			During the past 24 h, did the overall feeling of your oral cavity affect your well‐being ‘some/a lot/severely’?		
	Odds ratio	95% CI	*p*	Odds ratio	95% CI	*p*
Any oral symptom	27.09	12.87, 57.00	< 0.0001	12.1	6.22, 23.54	< 0.0001
Dry mouth	3.5	2.30, 5.34	< 0.0001	2.44	1.57, 3.81	< 0.0001
Oral pain	12.26	7.21, 20.83	< 0.0001	19.46	10.66, 35.52	< 0.0001
Thickening/swollen mucosa	3.57	2.02, 6.34	< 0.0001	3.75	2.05, 6.86	< 0.0001
Taste change	2.57	1.38, 4.76	0.0029	2.46	1.25, 4.81	0.0089
Mucosal sensitivity	3.85	2.02, 7.35	< 0.0001	2.93	1.50, 5.74	0.0017
Sticky saliva	2.31	1.17, 4.58	0.0163	2.75	1.33, 5.68	0.0063
Sensitive/tender teeth	10.58	4.02, 27.86	< 0.0001	11.48	4.16, 31.69	< 0.0001
Other oral symptoms	2.22	1.16, 4.24	0.016	1.77	0.91, 3.44	0.0947
Number of oral symptoms^a^	2.72	2.25, 3.28	< 0.0001	2.56	2.10, 3.12	< 0.0001

*Note:* Mixed effects logistic regression models modelling the probability of worse distress and impact on well‐being if oral symptoms were present. Models are adjusted for clinical site, regimen intensity (MAC vs. RIC/NMA), transplant type (allo vs. auto), sex, age, and days since HCT (first 17 days).

Abbreviations: HCT, haematopoietic cell transplantation; MAC, myeloablative conditioning regimen; NMA, non‐myeloablative conditioning regimen; RIC, reduced intensity conditioning regimen.

^a^ Number of symptoms is continuous; the estimate represents the effect of an additional symptom.

### 3.7 Symptom Burden Related to Transplant Type and Conditioning Regimen Intensity

Symptom burden did not differ between transplant types over the hospitalisation period (days 0 to 17) or during specific time intervals, except days 6 to 11. During this interval, patients receiving autologous HCT reported significantly worse well‐being than those undergoing allogeneic HCT (*p* = 0.0307). In terms of conditioning intensity, patients treated with MAC experienced worse distress due to the overall feeling of their mouth compared to patients treated with RIC/NMA (*p* = 0.0043) across the 0 to 17 days post‐HCT (Table 4). Similarly, a higher proportion of patients treated with MAC (56.6%) reported a moderate to severe impact on well‐being due to the overall feeling of their mouth compared to those treated with RIC/NMA (42.1% *p* = 0.0440). Interestingly, patients treated with MAC reported worse distress and well‐being from the overall feeling of the mouth compared to patients receiving RIC/NMA during intervals day 0 to 5 and 6 to 11 post‐HCT (Table 4). However, by day 12 to 17, no significant differences were seen in terms of level of distress or impact on well‐being. Both outcomes showed that approximately one‐third of patients were affected at a moderate to severe level.

**TABLE 4:** Distribution of patients responding some, a lot, or severe distress and some, a lot, or severe impact on their well‐being due to the overall feeling of the mouth.

		Total (Days 0–17)		Days 0–5		Days 6–11		Days 12–17	
		*N* (%)	*p*	*N* (%)	*p*	*N* (%)	*p*	*N* (%)	*p*
*Distress caused by the overall feeling of the mouth*									
MAC	Some/a lot/severely	64 (64.6)	0.0043	40 (41.2)	0.0011	50 (52.1)	0.0117	25 (28.7)	0.9392
RIC/NMA	Some/a lot/severely	42 (44.2)		17 (19.1)		30 (33.7)		24 (29.3)	
*Impact on well‐being caused by the overall feeling of the mouth*									
MAC	Some/a lot/severely	56 (56.6)	0.0440	32 (33.0)	0.0498	45 (46.9)	0.0051	26 (29.9)	0.6597
RIC/NMA	Some/a lot/severely	40 (42.1)		18 (20.2)		24 (27.0)		22 (26.8)	

*Note:* Questionnaire answers split by conditioning regimen intensity. *p*‐value compares MAC to RIC/NMA in each time interval after haematopoietic cell transplantation (HCT). *N* (%) refers to the number and the percentage of patients answering some/a lot/severely out of the total number of patients receiving MAC or RIC/NMA, respectively. The cohort consisted of 194 patients, of whom 99 patients received MAC and 95 patients received RIC/NMA.

Abbreviations: MAC, myeloablative conditioning; NMA, non‐myeloablative conditioning; RIC, reduced intensity conditioning.

## 4 Discussion

To the best of our knowledge, this is the first study to present a comprehensive view of the burden of oral symptoms in a large prospective cohort of adult allogeneic and autologous HCT recipients. The study shows that nearly all patients undergoing HCT experienced oral symptoms during the early post‐HCT phase, which caused both significant distress and a negative impact on overall well‐being. Oral symptoms were most prevalent and severe by day 6 to 11, as was the associated symptom burden. The majority of symptoms appeared earlier and more frequently in patients receiving MAC. Additionally, patients treated with MAC reported a higher symptom burden related to the feeling of the mouth compared to those treated with RIC/NMA during the first 11 days post‐HCT. By day 12, symptom burden was similar between the groups.

The overall incidence of oral symptoms was high, and the majority of patients experienced multiple symptoms during early post‐HCT hospitalisation. This underscores the clinical importance of effective oral symptom management. In a paediatric cohort of 68 patients undergoing allogeneic HCT, a total of 87% reported a range of oral symptoms (Agholme et al. 2023). Previous studies in adults have mainly investigated isolated oral symptoms such as oral mucositis‐related pain, dry mouth (xerostomia) and dysgeusia. A recently published study explored oral symptoms in patients with AML undergoing allogeneic HCT (Wysocka‐Słowik et al. 2021). Consistent with our findings, xerostomia was the most frequent symptom, followed by oral pain, burning mouth, and dysgeusia. Larsen et al. investigated self‐reported symptoms using repeated measures in the early phase after HCT in a mixed cohort of 43 patients. Mouth dryness, taste changes, pain, and mouth sores were frequently reported, particularly during the neutropenic phase (Larsen et al. 2004). Our findings confirm previously reported symptoms while providing a more detailed and comprehensive review as a result of frequent early post‐HCT assessments in a large cohort of patients with diverse diagnoses and treatment regimens.

Xerostomia and oral pain related to mucositis have, in some studies, been described by patients as the most debilitating symptoms after HCT (Bellm et al. 2000; Larsen et al. 2004). In the present study, xerostomia was the most frequently reported symptom and appeared early (within the first days post‐HCT) in many patients. However, some patients may have entered HCT with xerostomia. Regardless, the early occurrence of xerostomia may have contributed to the development of other interrelated oral symptoms such as sticky saliva, mucosal sensitivity and taste changes, thereby increasing the overall symptom burden. Hence, interventions aimed at treating or reducing xerostomia may have beneficial effects on the total symptom experience and associated burden. In the current study, oral pain was identified as the second most frequently reported symptom. Patients rated oral pain as the most bothersome on the NRS. However, oral pain may be underreported due to the use of effective analgesic medications. In addition, the use of cryotherapy for prevention of oral mucositis, as recommended in guidelines by the Multinational Association of Supportive Care in Cancer/MASCC (Keefe et al. 2007; Lalla et al. 2014) may have further reduced the reporting of oral pain.

Oral symptom frequency peaked on days 6 to 11 post‐HCT. This coincided with the highest symptom scores (NRS), greatest symptom burden and the lowest mean WBC and ANC. Similar temporal relations have been reported for isolated oral symptoms such as oral mucositis‐related pain (Agholme et al. 2023; Blijlevens et al. 2008), taste changes (Ferreira et al. 2020) and xerostomia (Bulthuis et al. 2023). Previous studies have associated symptom distress and reduced well‐being with three of the most common oral symptoms: oral pain related to oral mucositis (Guberti et al. 2022; Staudenmaier et al. 2018), dry mouth (Larsen et al. 2004) and taste changes (Scordo et al. 2022). The present study shows that all separate oral symptoms were associated with increased odds of greater distress and reduced well‐being due to the overall feeling of the mouth. Oral pain was the individual symptom that had the strongest impact on distress and overall well‐being. Furthermore, we demonstrated that each additional oral symptom increased the symptom burden, underscoring the importance of comprehensive symptom assessment and appropriate symptom management.

Transplantation type and conditioning intensity influenced symptom prevalence and the burden of oral symptoms in our study. Overall, patients (both autologous and allogeneic transplant) treated with MAC regimens reported oral symptoms earlier and more frequently compared to those receiving RIC/NMA. Similarly, Pereira et al. showed that oral alterations after HCT appeared early in patients undergoing autologous HCT and among those receiving myeloablative high‐dose melphalan conditioning, while they observed a later onset of oral alterations in allogeneic HCT recipients (Pereira et al. 2025). More specifically, in the present study, patients undergoing allogeneic HCT with MAC experienced the highest symptom frequencies throughout the observation time. In alignment with symptom prevalence, all patients treated with MAC reported a higher symptom burden on days 0 to 11 post‐HCT regardless of transplant type. These differences were not apparent by days 12 to 17, which may be due to the overall decrease of symptoms in autologous HCT recipients. The reasons for the prolonged duration of oral symptoms in patients undergoing allogeneic compared to autologous HCT remain unanswered, although differences between the groups (e.g., use of prophylaxis against graft‐versus‐host disease and TBI) may partially explain at least some of the observed differences. To our knowledge, no studies have specifically compared the impact of conditioning intensity on the burden of oral symptoms in HCT recipients. However, other studies have shown that non‐oral physical and psychological symptoms can significantly reduce quality of life early after HCT regardless of conditioning intensity (Newcomb et al. 2023). El‐Jawahri et al. (2015) reported comparable declines in quality of life across autologous HCT, allogeneic MAC, and RIC, suggesting that reduced‐intensity conditioning does not necessarily lessen symptom burden (El‐Jawahri et al. 2015).

The strengths of this study include its prospective, comprehensive, and longitudinal design. The inclusion of various medical diagnoses requiring transplantation, conditioning regimen, and transplant types allowed an overall analysis of symptoms and symptom burden in HCT recipients. However, there are challenges in repeatedly measuring patient‐reported outcomes in patients debilitated by disease or treatment.

The present study has several limitations. Analyses in this study were limited to days 0 to 17 post‐HCT. We recognise that many patients continue to experience symptoms beyond this period. A recent study showed that patients may have oral symptoms up to two years following allogeneic HCT (Leinbach et al. 2025). Furthermore, we acknowledge that patients with severe side effects and a high symptom burden may be unable or too weak to report their status or complete study questionnaires. This may potentially have led to an underestimation of symptoms. In spite of these limitations, the robust dataset enabled us to draw reliable conclusions about symptoms and symptom burden during the early post‐HCT hospitalisation period.

In conclusion, HCT recipients experienced multiple treatment‐related oral symptoms in the early post‐transplant phase which caused significant distress and negatively affected overall well‐being. MAC patients reported particularly high levels of oral symptoms. Many of these symptoms can be alleviated with supportive care and a proactive approach to symptom management may help improve patients' well‐being. Patients should be informed about the oral symptoms they may experience early after HCT, be instructed in self‐management strategies, and offered effective interventions for symptom relief.

## Author Contributions

**Kristina Skallsjö:** writing – original draft, funding acquisition, investigation, visualization, data curation, validation. **Michael T. Brennan:** conceptualization, investigation, writing – review and editing, project administration, supervision, resources, data curation, methodology, validation, software. **Bengt Hasséus:** investigation, writing – review and editing, supervision, methodology, validation. **Jenny Öhman:** writing – review and editing, supervision, investigation. **Judith E. Raber‐Durlacher:** investigation, writing – review and editing, validation. **Marie‐Charlotte D. N. J. M. Huysmans:** writing – review and editing, investigation, validation. **Alexa M. G. A. Laheij:** investigation, writing – review and editing, validation. **Stephanie J. M. van Leeuwen:** investigation, writing – review and editing, validation. **Allan J. Hovan:** writing – review and editing, investigation, validation. **Karin Garming Legert:** investigation, writing – review and editing, validation. **Scott Isom:** writing – review and editing, formal analysis, software, visualization. **David M. Kline:** writing – review and editing, formal analysis, software, visualization. **Nicole M. A. Blijlevens:** writing – review and editing, methodology, investigation, validation, supervision. **Jan‐Erik Johansson:** investigation, writing – review and editing, methodology, supervision, validation. **Inger von Bültzingslöwen:** conceptualization, investigation, validation, methodology, writing – review and editing, project administration, data curation, supervision, resources, software.

## Ethics Statement

This study was performed in accordance with the ethical guidelines of the Declaration of Helsinki. Approval was obtained from the Ethical Review Boards at each study site. Sweden: Regional Ethical Review Board in Gothenburg (513–10, T939‐16); Charlotte: Wake Forest School of Medicine Institutional Review Board (IRB00080071); Vancouver: BC Cancer Agency Research Ethics Board (BCCA REB # H11‐02350, BCCA REB # H15‐02350); Amsterdam and Nijmegen: Medical Ethical Research Committee, Amsterdam University Medical Center location AMC (NL52117.018.15), registered in the Dutch Trial Register (NL 5645). The approval granted in Amsterdam was validated by the IRB of Radboud UMC in Nijmegen.

## Conflicts of Interest

The authors declare no conflicts of interest.

## Supporting information


**Table S1:** Patient‐reported symptoms reported as the worst oral symptom by days after haematopoietic cell transplantation (HCT).

**Table S2:** Oral symptoms after HCT, days 0 to 17, by transplant type and conditioning intensity. Symptoms with a prevalence of 15% or lower are not displayed.

## Data Availability

Research data cannot be shared publicly because all data are stored at and belong to the University of Gothenburg (GU), and there are ethical and legal restrictions on sharing our data set according to Swedish law. Study data contain sensitive patient information that can be connected to individual patients. It may be possible to identify and connect personal information from the data set, even though data is de‐identified. All relevant data within the paper are at group level.
