# Supplementary material for: Patient‐Reported Oral Symptoms and Their Impact on Well‐Being After Haematopoietic Cell Transplantation
Source: Oral Dis. 2025 Sep 18;32(2):569–79. doi: 10.1111/odi.70099 (PMC13077019; doi:10.1111/odi.70099)
Supplement: Supplementary file 1 — Table S1: Patient‐reported symptoms reported as the worst oral symptom by days after haematopoietic cell transplantation (HCT). [file ODI-32-569-s001.docx]

| **Table S1** Patient-reported symptoms reported as the worst oral symptom by days after haematopoietic cell transplantation (HCT) | | | | |
| --- | --- | --- | --- | --- |
| Worst oral symptom,  n (%) | Total (Days 0–17)  N=194 | Days 0–5  N=189 | Days 6–11  N=186 | Days 12–17  N=173 |
| Dry mouth | 130 (67.0%) | 87 (46.0%) | 81 (43.5%) | 64 (37.0%) |
| Oral pain | 52 (26.8%) | 16 (8.5%) | 40 (21.5%) | 25 (14.5%) |
| Taste change | 32 (16.5%) | 13 (6.9%) | 21 (11.3%) | 18 (10.4%) |
| Thickening/swollen mucosa | 31 (16.0%) | 14 (7.4%) | 19 (10.2%) | 8 (4.6%) |
| Mucosal sensitivity | 22 (11.3%) | 8 (4.2%) | 15 (8.1%) | 4 (2.3%) |
| Sticky saliva | 19 (9.8%) | 8 (4.2%) | 11 (5.9%) | 5 (2.9%) |
| Sensitive/tender teeth | 9 (4.6%) | 1 (0.5%) | 5 (2.7%) | 4 (2.3%) |
| Mucosal coating | 4 (2.1%) | 3 (1.6%) | 2 (1.1%) | 0 (0.0%) |
| Easy bleeding from gums/oral mucosa | 2 (1.0%) | 0 (0.0%) | 2 (1.1%) | 1 (0.6%) |
| Other oral symptoms^*^ | 34 (17.5%) | 12 (6.3%) | 17 (9.1%) | 14 (8.1%) |
| Only one symptom could be listed as the worst at each visit, but patients could have multiple visits in each time interval.  ^*^Other oral symptom, unspecified. | | | | |
