# Supplementary material for: Patient‐Reported Oral Symptoms and Their Impact on Well‐Being After Haematopoietic Cell Transplantation
Source: Oral Dis. 2025 Sep 18;32(2):569–79. doi: 10.1111/odi.70099 (PMC13077019; doi:10.1111/odi.70099)
Supplement: Supplementary file 2 — Table S2: Oral symptoms after HCT, days 0 to 17, by transplant type and conditioning intensity. Symptoms with a prevalence of 15% or lower are not displayed. [file ODI-32-569-s002.docx]

| **Table S2**Oral symptoms after HCT, days 0–17, by transplant type and conditioning intensity. Symptoms with a prevalence of 15% or lower are not displayed. | | | | | | | | |
| --- | --- | --- | --- | --- | --- | --- | --- | --- |
| **Days after HCT** | **Total**  **(Days 0–17)** | **Day 0–5** | **Day 6–11** | **Day 12–17** | **Total**  **(Days 0–17)** | **Day 0–5** | **Day 6–11** | **Day 12–17** |
| **Allogeneic MAC** | **N=29** | **N=29** | **N=29** | **N=25** | **Allogeneic MAC vs Autologous MAC** | | | |
| Any oral symptom | 29 (100.0) | 26 (89.7) | 26 (89.7) | 21 (84.0) | **0.0127** | **0.0040** | 0.7108 | **<.0001** |
| Dry mouth | 27 (93.1) | 19 (65.5) | 20 (69.0) | 18 (72.0) | 0.2064 | 0.0701 | 0.8264 | **0.0011** |
| Oral Pain | 17 (58.6) | 9 (31.0) | 13 (44.8) | 10 (40.0) | **0.0002** | **<.0001** | 0.3558 | **<.0001** |
| Thickening/swollen mucosa | 19 (65.5) | 14 (48.3) | 13 (44.8) | 8 (32.0) | **<.0001** | **<.0001** | **0.0039** | **<.0001** |
| Taste change | 14 (48.3) | 8 (27.6) | 10 (34.5) | 10 (40.0) | **<.0001** | **0.0009** | **<.0001** | **<.0001** |
| Mucosal sensitivity | 12 (41.4) | 7 (24.1) | 8 (27.6) | 5 (20.0) | 0.0109 | 0.0886 | 0.0982 | **0.0002** |
| Sticky saliva | 16 (55.2) | 6 (20.7) | 11 (37.9) | 8 (32.0) | **<.0001** | **<.0001** | **0.0409** | **<.0001** |
| Other symptom^*^ | 7 (24.1) | 4 (13.8) | 3 (10.3) | 3 (12.0) | 0.2081 | 0.6908 | 0.1604 | 0.8734 |
| Multiple oral symptoms (≥2) | 27 (93.1) | 19 (65.5) | 24 (82.8) | 16 (64.0) | **<.0001** | **<.0001** | **0.0186** | **<.0001** |
| Mean (sd) | 3.6 (1.9) | 2.2 (1.7) | 2.9 (2.0) | 2.5 (2.0) | **<.0001** | **<.0001** | **0.0047** | **<.0001** |
|  |  |  |  |  |  | | | |
| **Autologous MAC** | **N=70** | **N=69** | **N=68** | **N=63** | **Autologous MAC vs Allogeneic RIC/NMA** | | | |
| Any oral symptom | 66 (94.3) | 52 (75.4) | 63 (92.6) | 37 (58.7) | **0.0399** | **0.0021** | 0.5668 | **0.0036** |
| Dry mouth | 60 (85.7) | 43 (62.3) | 50 (73.5) | 32 (50.8) | **0.0019** | **0.0447** | **0.0005** | **<.0001** |
| Oral pain | 26 (37.1) | 7 (10.1) | 25 (36.8) | 8 (12.7) | 0.1325 | **0.0092** | 0.2627 | 0.4268 |
| Thickening/swollen mucosa | 23 (32.9) | 11 (15.9) | 18 (26.5) | 3 (4.8) | **0.0452** | **0.0033** | 0.5518 | **0.0446** |
| Taste change | 14 (20.0) | 10 (14.5) | 6 (8.8) | 1 (1.6) | **<.0001** | **0.0334** | **0.0136** | **0.0105** |
| Mucosal sensitivity | 19 (27.1) | 12 (17.4) | 12 (17.6) | 3 (4.8) | 0.0585 | **0.0004** | 0.8701 | **0.0253** |
| Sticky saliva | 18 (25.7) | 5 (7.2) | 15 (22.1) | 2 (3.2) | 0.1407 | 0.4173 | 0.0591 | 0.1263 |
| Other symptom^*^ | 22 (31.4) | 9 (13.0) | 16 (23.5) | 9 (14.3) | 0.0860 | 0.0011 | 0.6676 | 0.0694 |
| Multiple oral symptoms (≥2) | 50 (71.4) | 32 (46.4) | 41 (60.3) | 16 (25.4) | **0.0328** | **0.0025** | 0.0536 | 0.0780 |
| Mean (sd) | 2.2 (1.4) | 1.4 (1.1) | 2.0 (1.4) | 0.9 (0.9) | 0.1030 | **0.0016** | 0.3073 | **0.0401** |
|  |  |  |  |  |  | | | |
| **Allogeneic RIC/NMA** | **N=92** | **N=88** | **N=86** | **N=84** | **Allogeneic MAC vs Allogeneic RIC/NMA** | | | |
| Any oral symptom | 79 (85.9) | 57 (64.8) | 67 (77.9) | 55 (65.5) | **0.0001** | **<.0001** | **0.0094** | **<.0001** |
| Dry mouth | 69 (75.0) | 50 (56.8) | 41 (47.7) | 37 (44.0) | **0.0004** | **0.0066** | **<.0001** | **<.0001** |
| Oral pain | 25 (27.2) | 10 (11.4) | 16 (18.6) | 17 (20.2) | **<.0001** | **<.0001** | **0.0002** | **0.0006** |
| Thickening/swollen mucosa | 20 (21.7) | 6 (6.8) | 15 (17.4) | 9 (10.7) | **<.0001** | **<.0001** | **<.0001** | **<.0001** |
| Taste change | 32 (34.8) | 18 (20.5) | 19 (22.1) | 19 (22.6) | **0.0114** | **0.0090** | **0.0199** | **0.0260** |
| Mucosal sensitivity | 19 (20.7) | 2 (2.3) | 14 (16.3) | 6 (7.1) | **<.0001** | **<.0001** | **0.0423** | **0.0002** |
| Sticky saliva | 15 (16.3) | 10 (11.4) | 6 (7.0) | 7 (8.3) | **<.0001** | **<.0001** | **<.0001** | **<.0001** |
| Other symptom^*^ | 23 (25.0) | 5 (5.7) | 16 (18.6) | 10 (11.9) | 0.9491 | **0.0331** | 0.5214 | 0.5105 |
| Multiple oral symptoms (≥2) | 53 (57.6) | 29 (33.0) | 34 (39.5) | 34 (40.5) | **<.0001** | **<.0001** | **<.0001** | **<.0001** |
| Mean(sd) | 1.9 (1.4) | 1.1 (1.2) | 1.5 (1.4) | 1.3 (1.3) | **<.0001** | **<.0001** | **<.0001** | **<.0001** |
| Patients could report more than one event within each time interval. Data are presented as number of patients and percentages, unless otherwise specified. Rightmost columns show p-values for group comparisons, based on chi-square tests.  * Other oral symptom, unspecified  n = 3 autologous HCT recipients received RIC/NMA conditioning. HCT, haematopoietic cell transplantation; MAC, myeloablative conditioning; RIC, reduced-intensity conditioning; NMA, non-myeloablative conditioning. | | | | | | | | |
